# Long Noncoding RNA H19 in Digestive System Cancers: A Meta-Analysis of Its Association with Pathological Features

**DOI:** 10.1155/2016/4863609

**Published:** 2016-09-21

**Authors:** Yang Lin, Lijian Xu, Wei Wei, Xiaohui Zhang, Rongchao Ying

**Affiliations:** ^1^Department of General Surgery, The Second Affiliated Hospital of Nanjing Medical University, Nanjing 210011, China; ^2^Department of Gastroenterological Surgery, Hangzhou First People's Hospital, School of Clinical Medicine, Nanjing Medical University, Hangzhou 310006, China

## Abstract

Long noncoding RNA (lncRNA) H19 has been reported to be upregulated in malignant digestive tumors, but its clinical relevance is not yet established. The meta-analysis was to investigate the association between H19 expression and pathological features of digestive system cancers. The databases of PubMed, EMBase, Web of Science, CNKI, and WanFang were searched for the related studies. A total of 478 patients from 6 studies were finally included. The meta-analysis showed that the patient group of high H19 expression had a higher risk of poorly differentiated grade, deep tumor invasion (T2 stage or more), lymph node metastasis, and advanced TNM stage than the group of low H19 expression, although there was no difference between them in terms of distant metastasis. Therefore, the high expression of lncRNA H19 might predict poor oncological outcomes of patients with digestive system cancers.

## 1. Introduction

The digestive system consists of the gastrointestinal tract (the esophagus, stomach, intestine, and colorectum) plus the accessory organs of digestion (the pancreas, liver, and gallbladder). The high morbidity and mortality rate of digestive system cancers remain a critical health problem worldwide [1]. Early-stage digestive system cancers have a significantly higher survival rate than advanced cancers, in which tumor invasion and metastasis are common pathological features of poor prognosis. Molecular analyses of cancer cells in various stages of progression have revealed that genetic alterations accumulate during tumor progression and correlate with the clinical aggressiveness of cancer. In recent years, a number of studies have found that long noncoding RNAs (lncRNAs), which refer to a class of RNA transcripts of greater than 200 nucleotides without protein-coding function [2], are closely related to tumor progression [3]. Their possible mechanism of action involves regulation of tumor suppressor genes and oncogenes on multiple levels [4], including transcription and posttranscription modification. lncRNA H19, imprinted maternally expressed transcript H19, is localized in 11p15.5 and encodes a noncoding RNA of 2.3 kb [5]. Previous studies have shown that lncRNA H19 is upregulated and related to the cell proliferation and migration in a variety of cancers, including breast cancer [6], bladder cancer [7], pancreatic cancer [8], gallbladder cancer [9], and glioma [10]. To validate its clinical relevance as a biomarker or therapeutic target, it is necessary to investigate whether lncRNA H19 expression is associated with pathological features. The current study was to conduct a systematic review and quantitative meta-analysis of this association in digestive system cancers.

## 2. Methods

### 2.1. Literature Search Strategies

The databases of PubMed, EMBase, Web of Science, CNKI, and WanFang were searched from their establishment to June 15, 2016, for the related studies in Chinese or English, which reported the association between lncRNA H19 expression and pathological features in digestive system cancers. The literature search strategy involved a combination of keywords (“long non-coding RNA or lncRNA”, “H19”, “cancer or carcinoma or tumor or neoplasm”, and “pathology”). Besides, the references of obtained literature were also traced to identify additional relevant studies.

### 2.2. Inclusion and Exclusion Criteria

The collected studies were considered eligible if they met the following criteria: (1) the expression of lncRNA H19 was detected in digestive system cancers; (2) the study provided at least one of following pathological features: histological grade, tumor invasion depth, lymph node metastasis, and distant metastasis; (3) it should be a case-control or cohort study; (4) the method of detecting lncRNA H19 was restricted to reverse transcription polymerase chain reaction (RT-PCR) with or without sequencing of PCR products. Exclusion criteria were as follows: (1) reviews, case reports, meta-analysis, and duplicate publications (only the most recent or high quality studies were included); (2) the studies with inaccurate description of pathological features or with description not for all patients; (3) the studies focusing on the molecular structure and function of lncRNA H19.

### 2.3. Literature Screening and Data Extraction

Two investigators (Yang Lin and Wei Wei) independently collected the data, according to the inclusion and exclusion criteria, with disagreement resolved by consensus or by discussion with a third investigator (Xiaohui Zhang) before analysis occurred. Data extraction of literature was as follows: first author, publication date, country of origin, cancer type, total number of patients, number of patients in high and low H19 expression groups, the detection method, and the cut-off estimates for H19 levels.

### 2.4. Quality Assessment

The quality of included studies was evaluated by using Newcastle-Ottawa Scale standard [17], which included selection (4 points), comparability (2 points), and outcome (3 points) with a score range of 0–9. Two investigators (Yang Lin and Wei Wei) independently evaluated each article with disagreement resolved by consensus or by discussion with a third investigator (Xiaohui Zhang). All eligible studies were scored in Table 1, with a higher score indicative of better methodological quality.

### 2.5. Statistical Analysis

Odds ratios (ORs) with 95% confidence intervals (CIs) were estimated to evaluate the association between lncRNA H19 expression and the pathological features in digestive system cancers. Statistical analysis was performed using Stata statistical software version 12.0 (Stata Corporation, College Station, Texas, USA). Chi-square-based *Q* and *I* ^2^ tests were used to determine the heterogeneity among the included studies. If the *P* value was greater than 0.1 and the *I* ^2^-value was less than 50%, the heterogeneity among studies did not reach statistical significance, and the fixed-effects model was subsequently implemented. Conversely, the random-effects model was applied for the analysis. Subgroup analysis and sensitivity analysis were used to determine the sources of heterogeneity. The publication bias was assessed using Begg's funnel plot, and a *P* value less than 0.05 was considered as the presence of publication bias.

## 3. Results

### 3.1. Data Selection and Characteristics

**Figure 1:**
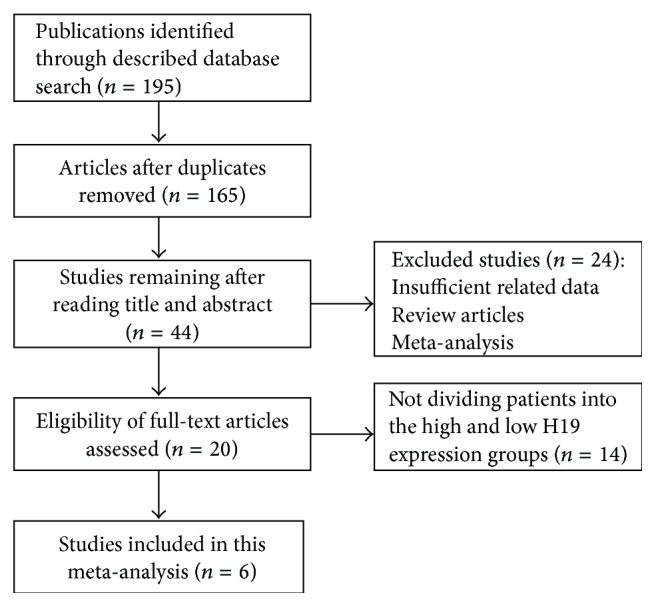
Flow chart of the study search and selection.

Six studies involving a total of 478 patients met the inclusion criteria. All studies came from China, in which three studies concentrated on gastric cancer, and the others involved esophageal cancer, colorectal cancer, and gallbladder cancer. RT-PCR was applied in detection of lncRNA H19, according to which the patients were categorized into the groups of high and low H19 expression. The cut-off estimates for H19 expression were the mean, median, or fold change (high expression group: greater than a 3-fold increase; low expression group: no more than a 3-fold increase). The flow chart of the study search and selection was showed in Figure 1. The characteristics of the included studies were summarized in Table 1.

### 3.2. Association between lncRNA H19 Expression and Pathological Features

#### 3.2.1. Histological Grade

**Figure 2:**
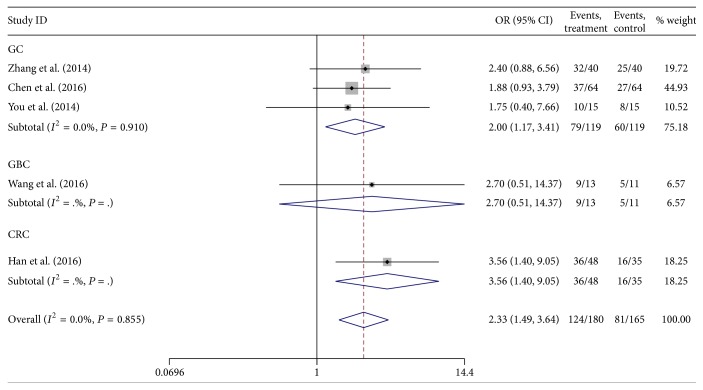
Forest plot for the association between lncRNA H19 expression and histological grade in digestive system cancers.

A total of five studies reported the association between lncRNA H19 expression and histological grade. The heterogeneity among studies was not statistically significant (*P* = 0.855, *I* ^2^ = 0.0%), so the fixed-effects model was applied to calculate the pooled OR and its 95% CI, which was significantly different [OR = 2.33, 95%  CI  (1.49,3.64), and *P* < 0.001] (Figure 2). It revealed that the group of high H19 expression had a higher risk of poorly differentiated grade than those of low H19 expression.

#### 3.2.2. Tumor Invasion Depth (T)

**Figure 3:**
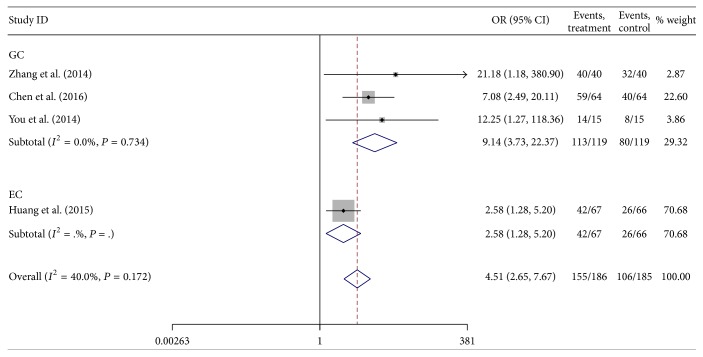
Forest plot for the association between lncRNA H19 expression and tumor invasion depth in digestive system cancers.

A total of four studies reported the association between lncRNA H19 expression and tumor invasion depth. The heterogeneity among studies was not statistically significant (*P* = 0.172, *I* ^2^ = 40.0%), so the fixed-effects model was applied to calculate the pooled OR and its 95% CI, which was significantly different [OR = 4.51, 95%  CI  (2.65,7.76), and *P* < 0.001] (Figure 3). It suggested that the group of high H19 expression had a higher risk of deep tumor invasion (T2 stage or more) than those of low H19 expression.

#### 3.2.3. Lymph Node Metastasis

**Figure 4:**
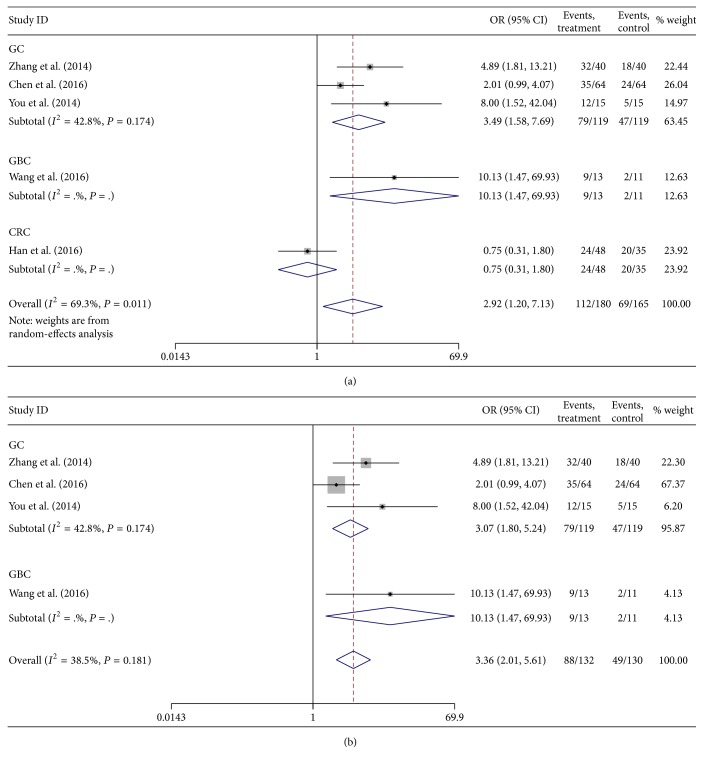
Forest plot for the association between lncRNA H19 expression and lymph node metastasis in digestive system cancers with (a) and without (b) including the work by Han et al.

A total of five studies reported the association between lncRNA H19 expression and lymph node metastasis. There was moderate heterogeneity among studies (*P* = 0.011, *I* ^2^ = 69.3%), so the random-effects model was applied to calculate the pooled OR and its 95% CI, which reached significant difference [OR = 2.92, 95%  CI  (1.20,7.13), and *P* = 0.018] (Figure 4(a)). Meanwhile, a subgroup analysis was performed by cancer types. One study by Han et al. [16] was omitted to measure its effect on the pooled OR. The heterogeneity among the four remaining studies was not statistically significant (*P* = 0.181, *I* ^2^ = 38.5%), so the fixed-effects model was applied to calculate the pooled OR and its 95% CI, which was significantly different [OR = 3.99, 95% CI (2.01,5.61), and *P* < 0.001] (Figure 4(b)). It demonstrated that the group of high H19 expression had a higher risk of lymph node metastasis than those of low H19 expression.

#### 3.2.4. Distant Metastasis

**Figure 5:**
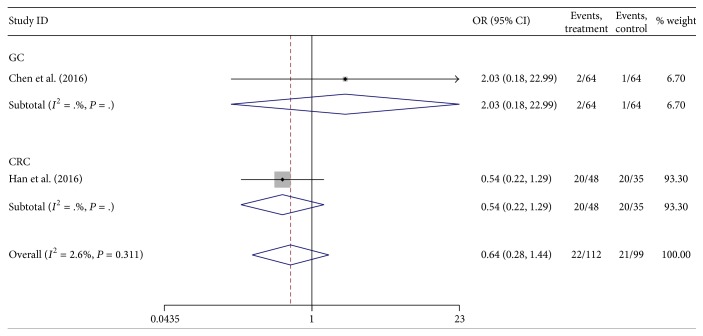
Forest plot for the association between lncRNA H19 expression and distant metastasis in digestive system cancers.

There were only two studies that reported the association between lncRNA H19 expression and distant metastasis. The heterogeneity among studies was not statistically significant (*P* = 0.311, *I* ^2^ = 2.6%), so the fixed-effects model was applied to calculate the pooled OR and its 95% CI, which did not reach significant difference [OR = 0.64, 95%  CI  (0.28,1.44), and *P* = 0.276] (Figure 5). It suggested that H19 expression levels were not correlated with distant metastasis.

#### 3.2.5. TNM Stage

**Figure 6:**
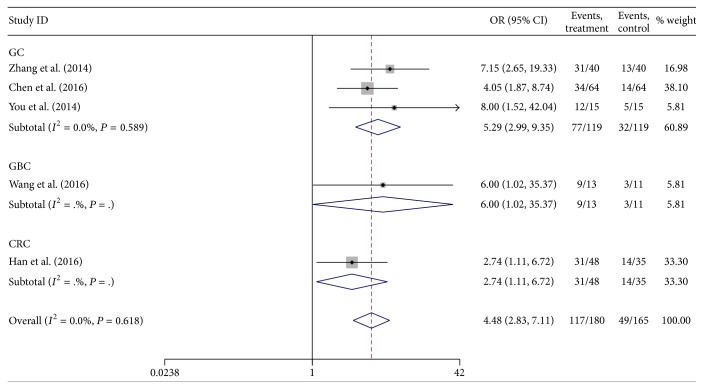
Forest plot for the association between lncRNA H19 expression and TNM stage in digestive system cancers.

There were five studies that reported the association between lncRNA H19 expression and TNM stage (III/IV versus I/II). The heterogeneity among studies was not statistically significant (*P* = 0.618, *I* ^2^ = 0.0%), so the fixed-effects model was applied to calculate the pooled OR and its 95% CI, which reached significant difference [OR = 4.48, 95%  CI  (2.83, 7.11), and *P* < 0.001] (Figure 6). It suggested that high H19 expression was linked with advanced TNM stage.

### 3.3. Assessment of Sensitivity Analysis

**Figure 7:**
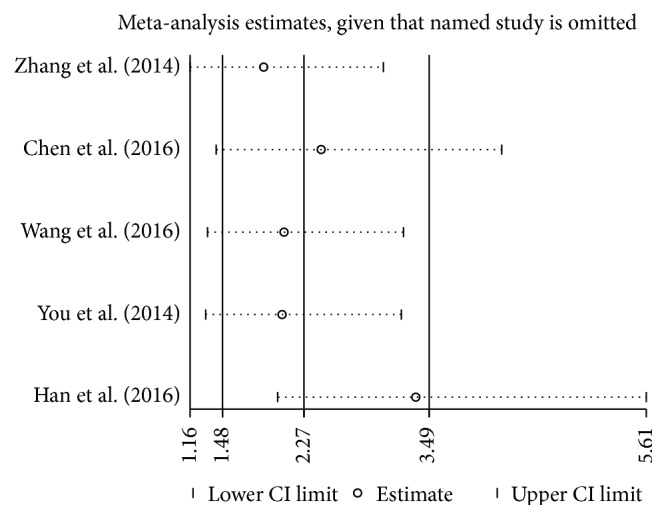
Sensitivity analysis of the included studies for the association between lncRNA H19 expression and lymph node metastasis.

Sensitivity analysis was performed to assess the effect of individual study on the pooled ORs for the association between H19 expression and lymph node metastasis (Figure 7). The main source of heterogeneity was the work by Han et al. [16]. When their study was excluded, the analytical result was not significantly altered. Similarly, sensitivity analysis was applied in investigation of other pathological features. As each study was omitted sequentially, the analytical results had not yet been significantly altered.

### 3.4. Assessment of Publication Bias

Begg's funnel plot was performed to assess publication bias in the study. There was no significant publication bias in analysis of histological grade (*P* = 0.772), lymph node metastasis (*P* = 0.226), and TNM stage (*P* = 0.331). Due to the small number of studies, publication bias was not analyzed in tumor invasion depth and distant metastasis.

## 4. Discussion

lncRNAs have been reported to play significant roles in carcinogenesis and progression of malignant tumors [15, 16, 18, 19]. Comparative analysis of lncRNA expression profiles between cancerous tissues of different stages has revealed that some lncRNAs are differentially expressed in association with the metastatic potential of cancer cells. They have been also identified as having functions of inducing or suppressing metastasis in experimental models, among which H19 is closely related to progression of digestive system cancers. High expression of H19 promoted the proliferation and invasion of esophageal cancer cell lines, whereas the opposing effects were observed by H19 knockdown [12]. In colorectal cancer, H19 acted as miRNA sponges to restore activation of multiple oncogenes, which promoted epithelial-to-mesenchymal transition (EMT) and were inherently suppressed by miR-138 and miR-200a [20]. Furthermore, elevated expression of H19 activated miR-194-5p targeting AKT2 gene expression by downregulating miR-194-5p and stimulated proliferation of gallbladder cancer cells by promoting cells into S-phase [9]. H19 and its mature product miR-675 enhanced the proliferation and invasion of gastric cancer AGS cells by activation of Akt/mTOR pathway, in which tumor suppressor RUNX1 served as a pivotal mediator [21]. The recent studies have indicated that overexpression of H19 also promoted EMT progression in EC, GC, GBC, and CRC [12–14, 20] in vitro, which suggested that lncRNA H19 might play an important role in invasiveness and metastasis, as a biomarker for prognosis of digestive system cancers.

The current meta-analysis was to investigate the association between lncRNA H19 expression and pathological features in digestive system cancers. A total of 478 patients from 6 studies were finally enrolled. The fixed-effects model was applied for histological grade, tumor invasion depth, distant metastasis, and TNM stage. Since there was moderate heterogeneity among the studies in terms of lymph node metastasis (*P* = 0.011, *I* ^2^ = 69.3%), the random-effects model was used to pool data. As a result, the group of high H19 expression had a higher risk of poorly differentiated grade, deep tumor invasion, lymph node metastasis, and advanced TNM stage than those of low H19 expression, although there was no significant difference in terms of distant metastasis (*P* = 0.276).

Then the subgroup analysis and sensitivity analysis were performed to investigate whether the heterogeneity of data took effect on interpretation of the analytic results. Consequently, the work by Han et al. [16] was the main source of heterogeneity among the studies of lymph node metastasis, and it was therefore omitted to assess its effect on the pooled OR. The heterogeneity among the rest of the studies was not statistically significant (*P* = 0.181, *I* ^2^ = 38.5%), and the result was not significantly altered. Besides, the assessment of publication bias did not reach statistical significance (*P* = 0.226).

However, there were some limitations in the study: (1) all of the studies came from China and did not include the patients of other countries; (2) the number of patients enrolled in some studies was relatively small, and not all cancer types of digestive system were under investigation; (3) there was no consensus on the cut-off estimates for differentiating high or low H19 expression; (4) no cohort studies met the inclusion criteria and were included. High quality and large sample-size studies are guaranteed to confirm the investigation outcomes.

In summary, the high expression of lncRNA H19 was linked with poorly differentiated grade, deep tumor invasion, and lymph node metastasis, suggesting it as a biomarker of poor prognosis for patients with digestive system cancers.

## Figures and Tables

**Table 1:** Characteristics of the included studies.

Authors	Year	Country	Cancer type	Total number	H19 expression		Cut-off (high/low)	Detection method	Quality score
					High	Low			
Zhang et al. [11]	2014	China	GC	80	40	40	Mean	RT-qPCR	7
Huang et al. [12]	2015	China	EC	133	66	67	Median	RT-qPCR	8
Chen et al. [13]	2016	China	GC	128	64	64	Median	RT-qPCR	8
Wang et al. [14]	2016	China	GBC	24	13	11	Median	RT-qPCR	6
You et al. [15]	2014	China	GC	30	15	15	Median	RT-qPCR	6
Han et al. [16]	2016	China	CRC	83	48	35	Fold change	RT-qPCR	7

GC: gastric cancer; EC: esophageal cancer; GBC: gallbladder cancer; CRC: colorectal cancer; RT-qPCR: real-time quantitative PCR.
